# Multidisciplinary Management of Aggressive Orbital Rhabdomyosarcoma in an Adult Patient: A Case Report and Literature Review

**DOI:** 10.7759/cureus.81828

**Published:** 2025-04-07

**Authors:** Leila Afani, Anas Baladi, Nazih Assabban, Yassine Benchamkha, Rhizlane Belbaraka

**Affiliations:** 1 Department of Medical Oncology, Centre Hospitalo-Universitaire (CHU) Mohammed VI, Marrakech, MAR; 2 Department of Plastic Surgery, Centre Hospitalo-Universitaire (CHU) Mohammed VI, Marrakech, MAR

**Keywords:** case report, chemotherapy, craniomaxillofacial surgery, orbit malignancy, rhabdomyosarcoma (rms)

## Abstract

Embryonic rhabdomyosarcoma (RMS) is a common tumor in children. In adults, it is an extremely rare tumor. There are three histological subtypes: embryonic, alveolar, and pleomorphic RMS. Pleomorphic RMS is most common in adults. Orbital site is a common primary site in children. In adults, it is an unusual presentation site.

Management of RMS is based on a multimodal approach, including surgery, radiotherapy, and chemotherapy. In the pediatric population, a multidisciplinary approach has been validated in prospective trials in Europe and the USA. In adults, there is no consensus on the management of these tumors in the absence of dedicated clinical trials. Management of adult RMS is based on pediatric guidelines and data from retrospective series of adults. The prognosis of RMS differs according to age. Adult RMS has worse survival than in children. We report a case of a 37-year-old female patient presenting with an orbital embryonic RMS. Treatment involved enucleation, but rapid local progression was observed. Chemotherapy was initiated, but evaluation after three cycles demonstrated significant tumor progression. Surgical intervention combined with radiotherapy was performed, followed by adjuvant chemotherapy. This case highlights the aggressive biology of embryonal RMS in adults and the role of a multimodal treatment approach.

## Introduction

Rhabdomyosarcoma (RMS) is a soft tissue sarcoma that originates from mesenchymal cells. It is the most common extracranial tumor in children after neuroblastoma and Wilms' tumor [1].

In adults, RMS is extremely rare [2]. A recent study identified only 50 cases reported between 1978 and 2018 [3]. In adults, the most common primary sites are the extremities, while head and neck RMS accounts for only 9% of cases. Orbital localization accounts for only 0.7% of cases in adults [4].

There are several histological subtypes of RMS. Embryonal and alveolar subtypes are specific to children. Pleomorphic RMS is most common in adults. This is associated with a poor prognosis [5].

The clinical presentation of orbital RMS is often a common orbital mass. The progression of RMS is generally rapid. Diagnosis is made via MRI, which assesses the tumor's location, involvement of surrounding soft tissues, and potential intracranial extension [6]. Final diagnosis is confirmed through excisional or incisional biopsy with immunohistochemical analysis [7].

Due to the rarity of RMS in adults, no standardized treatment guidelines exist. Most available data come from published case series [2-4].

Treatment is based on a multidisciplinary approach involving surgery, radiotherapy, and chemotherapy [1]. Surgery alone is rarely an option for orbital RMS. It is often combined with radiotherapy and chemotherapy, depending on the risk group [7]. The prognosis for RMS in adults remains poor compared with that in children [4].

Here, we report a rare case of orbital embryonal RMS in an adult, with an unusual clinical presentation and histological subtype. This case also illustrates the aggressiveness of these tumors in adults and the need for standardized treatment guidelines in this population.

## Case presentation

A 37-year-old woman presented to the ophthalmologic department with unilateral redness and tearing of the right eye that had begun in January 2022. The symptoms worsened in August 2023, with rapid enlargement of the mass, increased pain, and new bleeding upon palpation.

**Figure 1:**
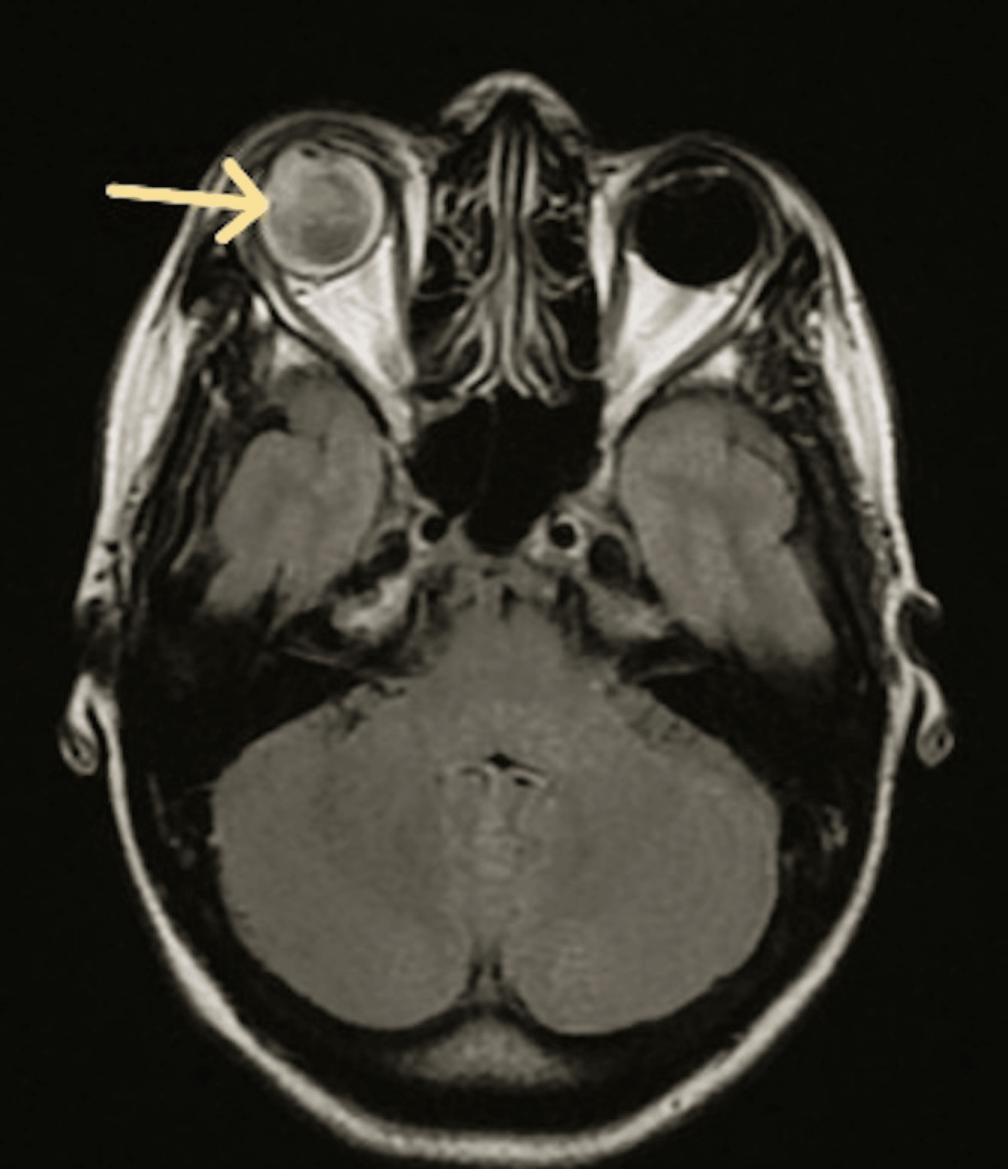
Cranial MRI revealing a vitreous hemorrhage with an intraocular mass in the anteroinferior quadrant, demonstrating marked periorbital fat infiltration.

MRI angiography of the orbit, performed in September 2023, revealed vitreous hemorrhage associated with an intraocular mass located in the anteroinferior region, with significant infiltration of periorbital fat (Figure 1).

In September 2023, the patient underwent emergency enucleation. The surgical specimen contained the eyeball. It was ruptured and fragmented.

**Figure 2:**
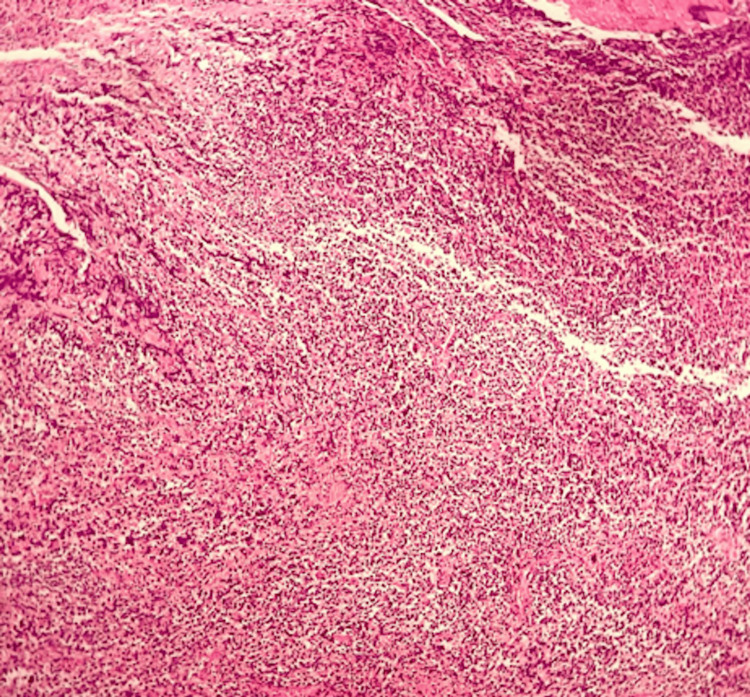
Microscopic features showing tumor proliferation largely flattened (hematoxylin and eosin, x100).

**Figure 3:**
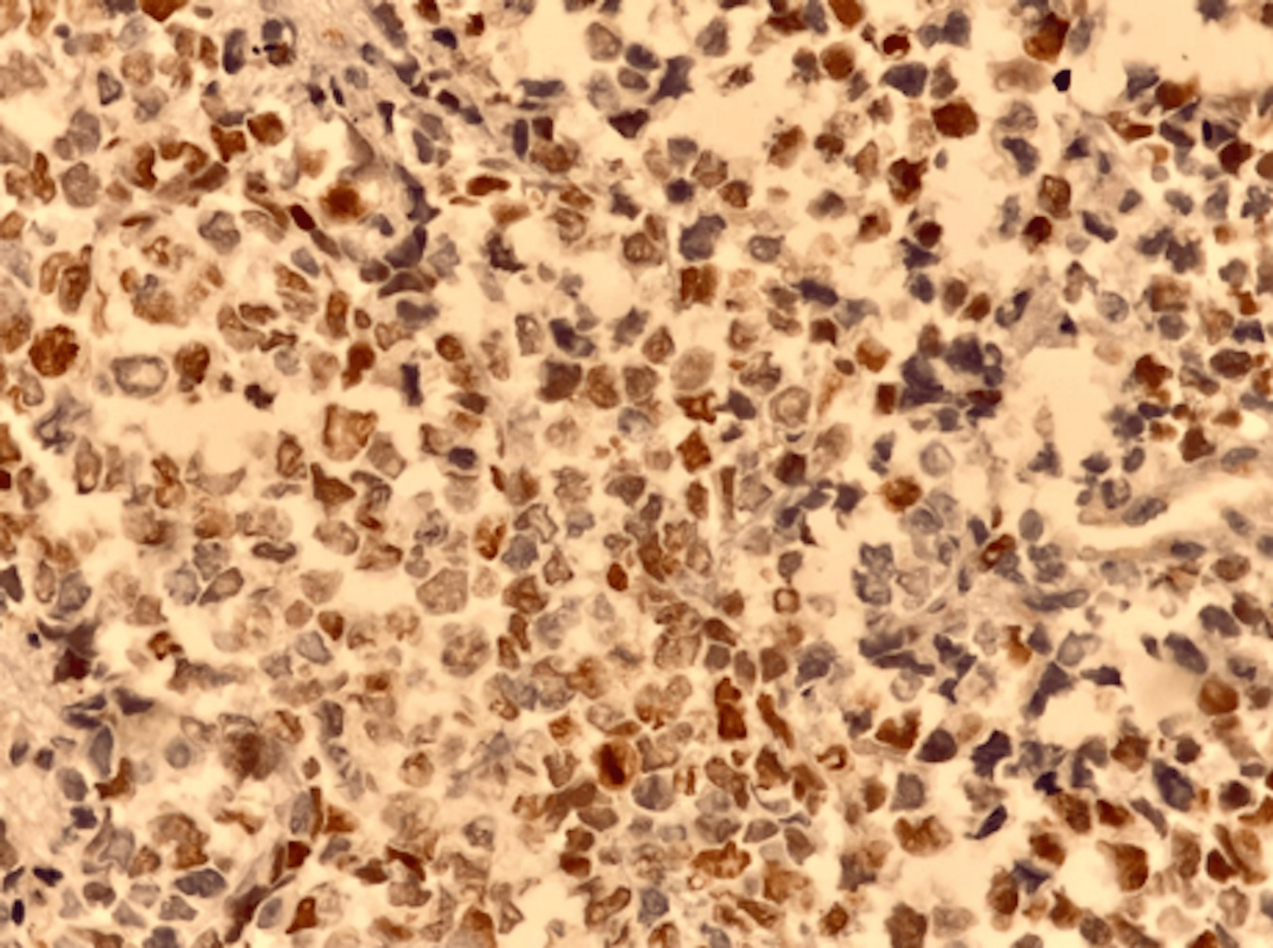
Microscopic features showing positive nuclear staining of tumor cells by anti-MyoD1 antibody (x400).

Histological examination demonstrated malignant proliferation of large cells, initially suggestive of lymphoma. Immunohistochemical analysis showed positivity for TdT, desmin, myogenin, and MyoD1 antibodies, confirming embryonal rhabdomyosarcoma (Figures 2, 3).

**Figure 4:**
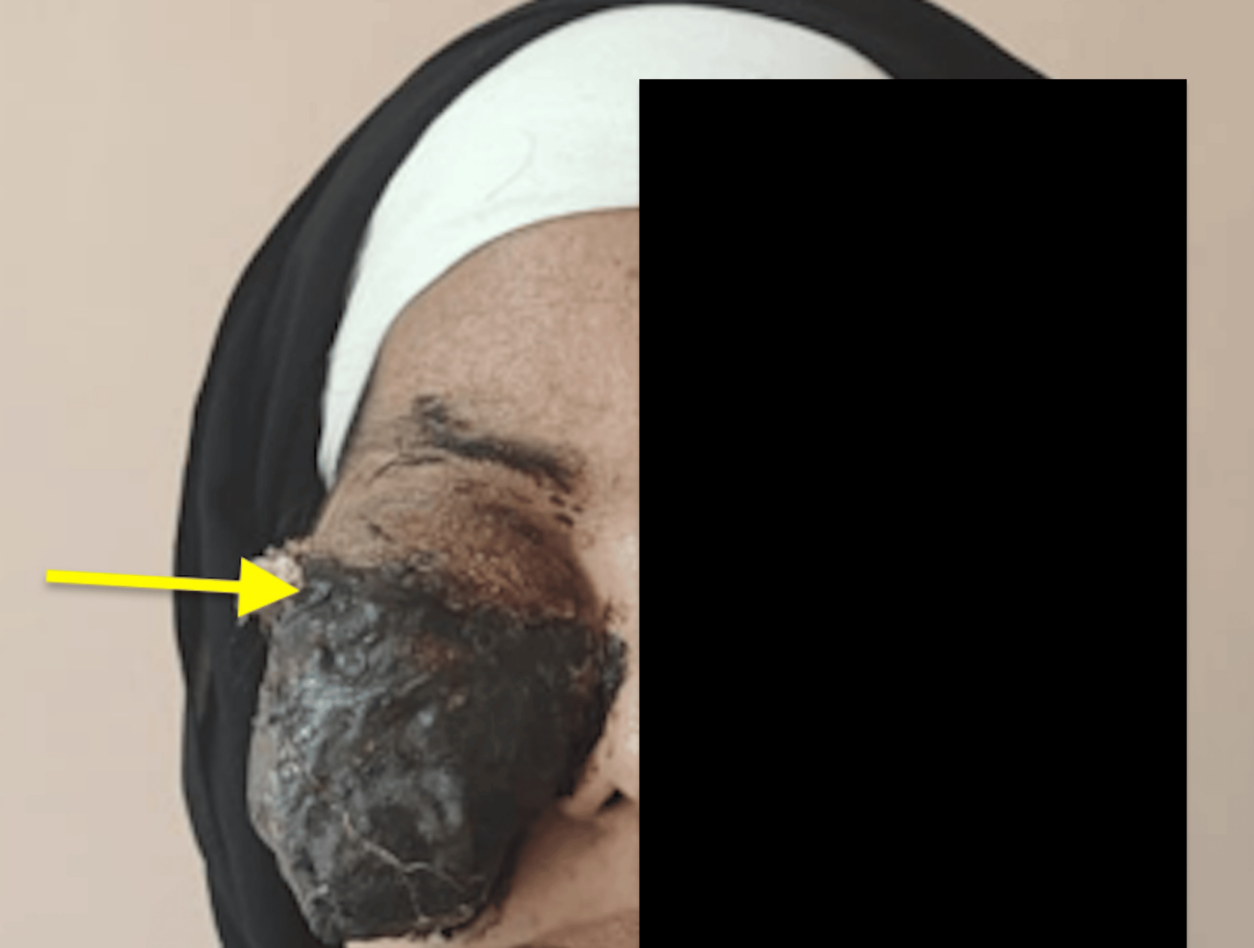
Photo showing a large necrotic mass in the orbital region (yellow arrow).

Two months later, a necrotic mass appeared in the right orbital region (Figure 4).

**Figure 5:**
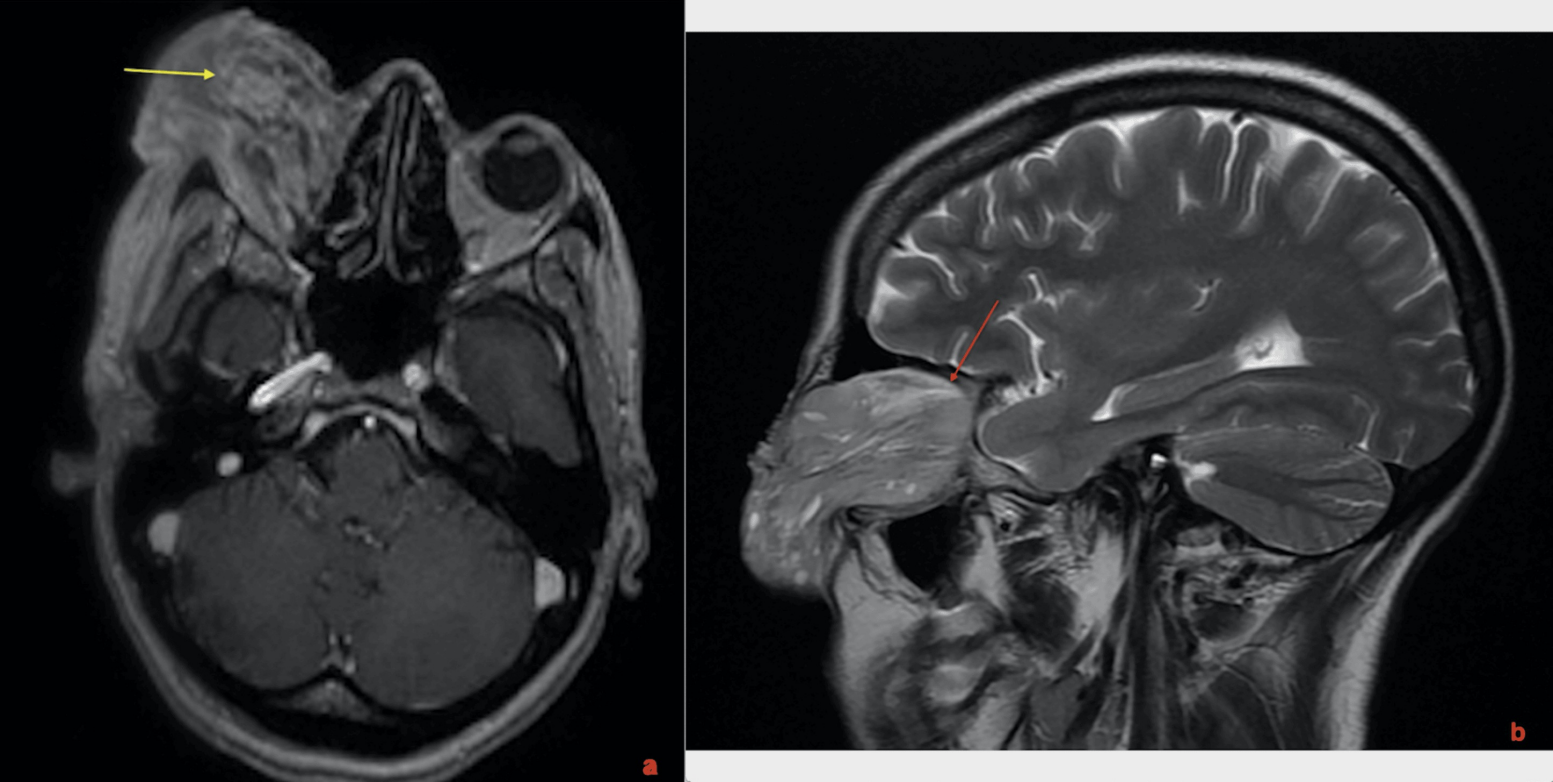
Orbital and cranial MRI showing a large mass involving the globe, muscles, lacrimal gland, and extraconal fat (yellow arrow in figure (a)), with initial intracranial infiltration (red arrow in figure (b)).

The orbital and cranial MRI showed significant progression of right orbital lesions involving the globe, muscles, lacrimal gland, and extraconal fat, with initial intracranial infiltration (Figure 5).

A CT scan of chest and abdominal imaging did not show any secondary localizations. The clinical case was discussed in a multidisciplinary team meeting. Given the rapid progression of the tumor and its high volume, induction chemotherapy was indicated.

**Figure 6:**
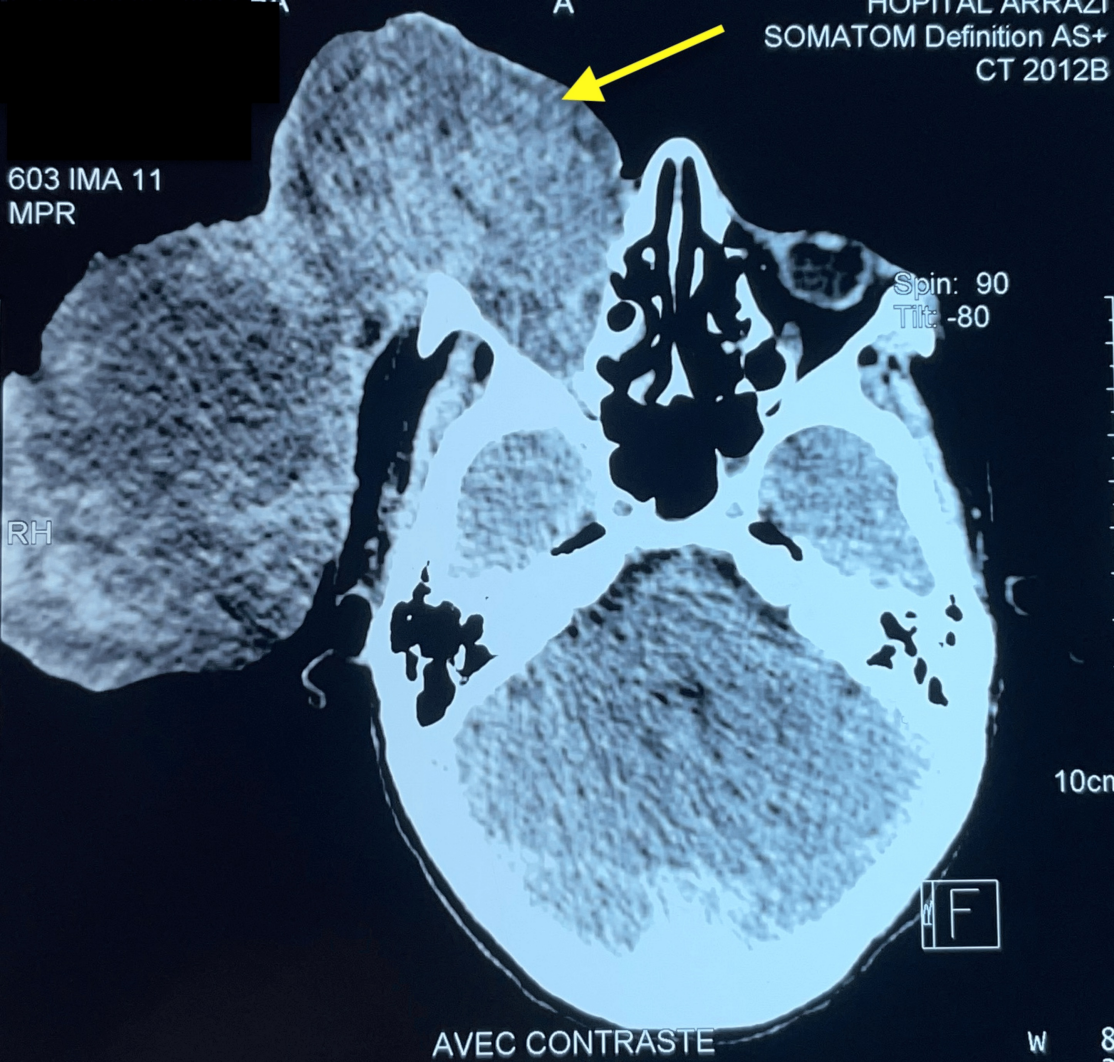
The orbital and cranial CT scan showed a large tumor process of the right eyeball measuring 18 x 12 x 14 cm, after chemotherapy (yellow arrow).

In November 2023, the patient received three cycles of VAC (vincristine, actinomycin, cyclophosphamide) chemotherapy, but the orbital mass continued to progress. This was associated with a hemorrhagic syndrome and a critically low hemoglobin level of 3 g/d, requiring emergency hospitalization for transfusion. It should be noted that the platelet count and hemostasis assessment were normal. So the hemorrhagic syndrome was not due to chemotherapy toxicity. The orbital and cranial CT scan, performed in January 2024, showed a large tumor process of the right eyeball measuring 18 x 12 x 14 cm. The tumor extends externally and involves both extra and intraconal orbital fat without intracranial extension (Figure 6).

**Figure 7:**
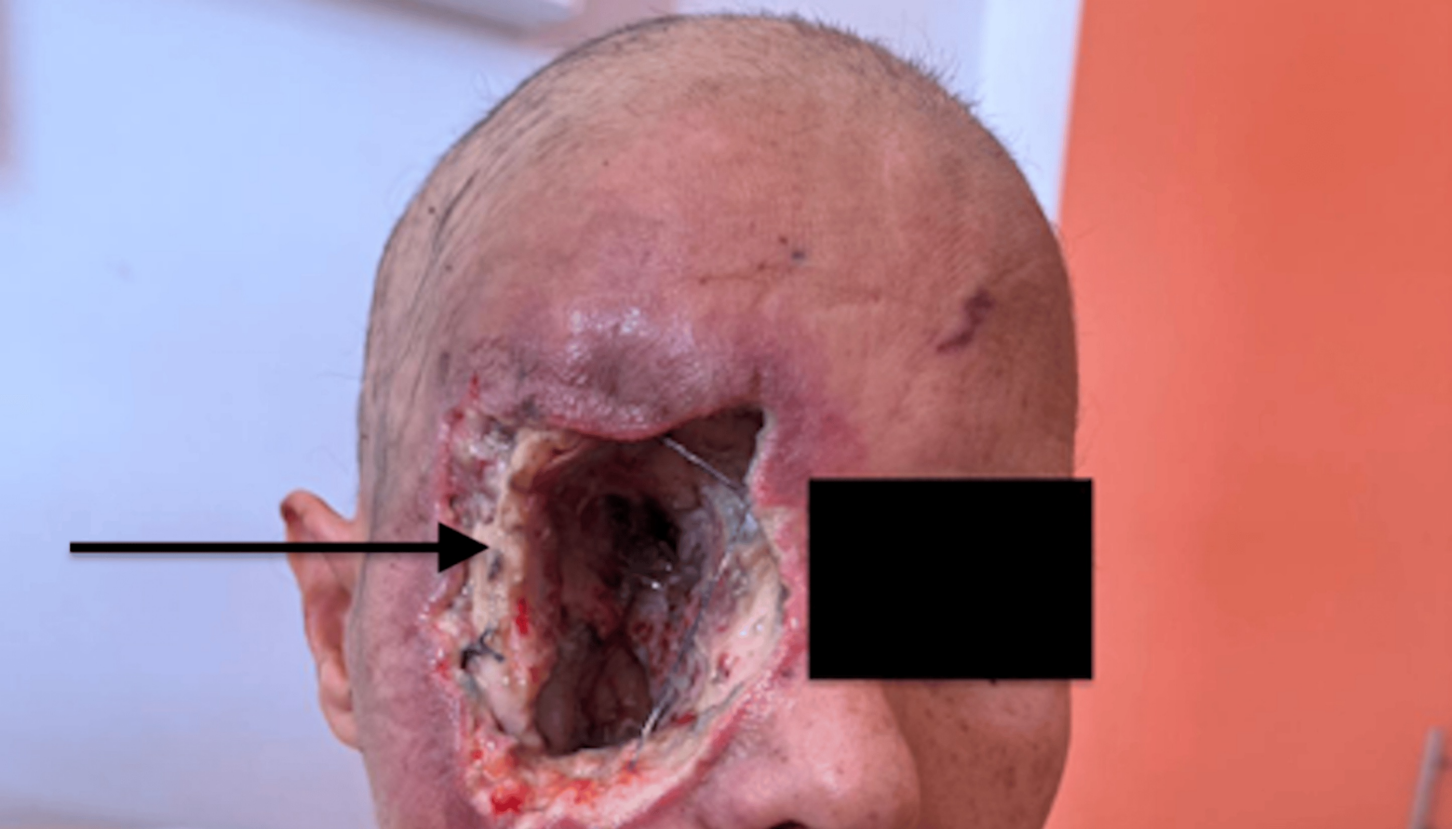
Photo showing the orbital cavity after wide resection (black arrow).

Due to disease progression despite chemotherapy, a wide resection was performed in January 2024 (Figure 7), followed by adjuvant radiotherapy.

The patient received adjuvant radiation therapy at a dose of 50 Gy over five weeks, starting in late February and concluding in March 2024. In April 2024, the patient underwent three additional cycles of chemotherapy with a regimen based on ifosfamide, vincristine, and actinomycin.

At six months post treatment, the patient remains stable with no radiologic evidence of recurrence. Table 1 provides a summary of the treatment phases. However, ongoing long-term surveillance is necessary due to the high risk of recurrence associated with adult RMS.

**Table 1:** Summary of treatments. RMS: rhabdomyosarcoma; VAC: vincristine, actinomycin, and cyclophosphamide.

Date	Treatment	Response/outcome
Sep 2023	Emergency enucleation	Initial control, but recurrence observed
Nov 2023	Diagnosis confirmation (RMS) + mass recurrence	Tumor progression despite VAC
Nov 2023 – Jan 2024	VAC chemotherapy (three cycles: vincristine, actinomycin D, cyclophosphamide)	No response; hemorrhagic syndrome, anemia (hemoglobin = 3 g/dL)
Jan 2024	Wide surgical resection	Large residual tumor removed
Feb – Mar 2024	Adjuvant radiotherapy (50 Gy over five weeks)	Stabilization of local disease
Apr 2024 – Jun 2024	Second-line chemotherapy (three cycles: ifosfamide, vincristine, actinomycin)	No recurrence at six-month follow-up

## Discussion

RMS is a common pediatric tumor, accounting for more than 50% of soft tissue sarcomas in children and adolescents [1]. In adults, however, it is rare. Soft tissue sarcomas represent less than 1% of adult solid tumors, with RMS comprising less than 3% of these sarcomas [2].

Orbital localization is frequent in pediatric cases, representing 10-20% of all RMS in children [6]. In a recent analysis of the Surveillance, Epidemiology, and End Results (SEER) database, including 1,071 adults treated for RMS, the most common sites were the extremities, trunk, and genitourinary system, with orbital localization accounting for only 0.7% of cases in adults [4].

The primary clinical signs of orbital RMS include proptosis, globe displacement, blepharoptosis, and a palpable mass. Pain is reported in only 10% of cases [6]. RMS progression is typically rapid [6]. Orbital RMS invades bone and extends into intracranial cavities. Lymph node involvement is rare, and metastatic dissemination is uncommon. The most common metastasis occurs in the lung, followed by bone [7].

Imaging plays a key role in diagnosing orbital RMS [6]. CT scans provide better evaluation of bony structures, while MRI is superior for characterizing the tumor's location, soft tissue involvement, and intracranial extension [6]. In this case, MRI findings of intracranial infiltration prompted aggressive multimodal therapy. Orbital RMS is often classified as an extraconal lesion. On T1-weighted sequences, the lesion is isointense with extraocular muscles. On T2-weighted sequences, the lesion typically appears hyperintense relative to extraocular muscles and orbital fat [8].

The staging of orbital RMS includes a thoracic CT and bone scintigraphy. PET-CT is preferred for better staging of high-risk soft tissue sarcomas [9]. Diagnosis is confirmed through excisional or incisional biopsy if complete resection is not feasible.

RMS has three main histological subtypes: embryonal, alveolar, and pleomorphic. The embryonal subtype includes botryoid and spindle cell variants, which exhibit different growth patterns and prognostic implications. This subtype is common in children and adolescents [5]. In adults, pleomorphic RMS predominates [6].

Embryonal RMS is characterized by spindle and round cells exhibiting features of skeletal muscle at various stages of embryogenesis [6].

Immunohistochemistry (IHC) aids in diagnosing RMS. Commonly used markers include antibodies against desmin, muscle-specific actin, and myoglobin. Vimentin is typically positive but not specific to RMS [7]. In adults, the differential diagnosis includes other malignant extraconal tumors. Orbital lymphoma accounts for 10-15% of orbital tumors. Orbital metastases represent up to 13% of orbital tumors. Breast cancer is the most common primary site [10]. In the presented case, the clinical presentation, imaging, and initial histological study suggested a diagnosis of orbital lymphoma. The diagnosis of embryonal orbital RMS was ultimately confirmed through immunohistochemical analysis.

The treatment of RMS is based on a multimodal approach, including surgery, chemotherapy, and radiotherapy. There are no prospective data available for RMS in adults. Management of adult RMS is based on pediatric guidelines and data from retrospective series of adults [11].

Until 1960, the treatment for RMS consisted of orbital exenteration. This aggressive approach was abandoned with the introduction of chemotherapy and radiotherapy. Currently, surgery is limited to excisional biopsy only if visual and aesthetic functions can be preserved. Often, the tumor is large with posterior invasion, and complete resection cannot be achieved. In such cases, incisional biopsy is indicated [6]. In our clinical case, surgery was indicated initially due to active bleeding from the tumor.

RMS is divided into four prognostic groups according to the Intergroup Rhabdomyosarcoma Study classification. Group I defines resected localized disease. Group II refers to residual microscopic disease after biopsy. Group III involves residual macroscopic disease. Group IV refers to metastatic disease [7].

In orbital RMS, a complete surgical resection (R0) is rarely possible. Radiotherapy is the standard treatment for group II (microscopic residual) and group III (macroscopic residual) tumors. Radiotherapy is initiated six to 12 weeks after neoadjuvant chemotherapy. It is delivered at a dose of 36-50 Gy over four to five weeks [12]. Other radiotherapy techniques, such as intensity-modulated radiation therapy, brachytherapy, and proton beam therapy, can also be used. These techniques achieve a higher treatment dose without increasing toxicity [13]. Given the R2 resection, our patient was considered high-risk, and radiotherapy was indicated.

Chemotherapy has been introduced in RMS since 1960. Chemotherapy protocols for adults are derived from protocols used in pediatric departments. The VAC protocol is the standard in North American countries. In Europe, the IVA protocol (ifosfamide, vincristine, actinomycin) has shown similar results [14]. The VAC protocol is indicated in non-metastatic pediatric RMS with intermediate or high risk [15]. For low-risk patients, chemotherapy with vincristine and dactinomycin has the same five-year event-free survival (EFS) [16]. For high-risk patients, maintenance chemotherapy with oral cyclophosphamide and vinorelbine improves outcomes [17].

In a large retrospective study of 180 adult patients, 59 patients received chemotherapy, and the response rate to chemotherapy was 85% for patients with embryonal and alveolar RMS. Only nine patients progressed under chemotherapy. These patients were treated according to pediatric protocols [2].

In a retrospective study of 80 adult patients with RMS, 27 patients received chemotherapy. Protocols based on vincristine, cyclophosphamide, doxorubicin, or dactinomycin were used. The response rate was 75%. Only five patients had a non-response, and two patients progressed under chemotherapy. The 10-year metastasis-free survival was 72% among responders. The overall 10-year survival rate was 47% [18]. Our clinical case illustrates a chemoresistance uncommon to embryonal RMS, suggesting different tumor biology between adults and children. This case reinforces the need for further research into molecular differences in adult RMS, which may impact treatment resistance.

Literature data clearly show that multimodal management of RMS is essential to improve the prognosis of these tumors (Table 2). The prognosis of RMS depends on age, location, histological type, and stage [6]. Compared to children, the prognosis in adults is poor. According to a comparative study of 2600 patients, the five-year overall survival rate was 27% in adults compared to 61% in children [4]. A different tumor biology was mentioned in some studies. Adult RMS presents a pronounced expression of multidrug-resistance protein than children RMS [19]. In adults, the anatomical location of the tumor is often unfavorable. Also, intolerance to intensive treatments may explain this difference [4]. This case illustrates the challenges of treating aggressive orbital RMS in adults, emphasizing the need for tailored treatment strategies beyond pediatric protocols.

**Table 2:** Summary of key studies in adult rhabdomyosarcoma. HN: head and neck; GU: genitourinary; S: surgery; RT: radiotherapy; CMT: chemotherapy; OS: overall survival; DFS: disease-free survival; EFS: event-free survival; PFS: progression-free survival; NOS: not otherwise specified; y: year.

Series	Number of cases	Median age	Study period	Localization	Histology	Stage	Treatment	Results
Little et al. (2002) [18]	82	27	1960-1998	HN: 43. Orbital: 2. Extremity: 18. Trunk: 21. Others: 504	Embryonal: 28. Alveolar: 19. Pleomorphic: 35. NOS: 2	Stage IIB (T1N0): 26. Stage III (T2bN0): 25. Stage IV (any T,N1,M0): 27	S+RT+CMT: 37%. S+ RT: 18%. RT+CMT: 34%. RT alone: 11%	10-y OS: 40%. 10-y DFS: 41%
Ferrari et al. (2003) [2]	171	27	1975-2001	HN: 53. Extremity: 48. GU: 36. Others: 43	Embryonal: 60. Alveolar: 62. Pleomorphic: 37. NOS: 21	Localized: 149. Distant: 31	S: 40%. RT: 56%. CMT: 72.5%	5-y EFS: 27.9%. 5-y OS: 39
Sultan et al. (2009) [4]	1071	NR	1973-2005	HN: 100. Orbital: 8. Extremity: 281. GU: 178. Others: 504	Embryonal: 20%. Alveolar: 14%. Pleomorphic: 19.1%. NOS: 43.3%	Localized: 339. Regional: 273. Distant: 302	S: 45%. RT: 41%. CMT: no data	5-y OS: 27%
Mäkinen et al. (2021) [3]	50	46.5	1979-2018	HN: 4. Extremity: 14. GU:15. Others: 7	Pleomorphic: 22. Others: 28	Localized: 30. Distant: 20	S: 73%. RT: 48%. CMT: 54%	5-y OS: 30%
Zhao et al. (2024) [13]	42	28	2008-2022	Orbital: 1. Parameningeal: 26. Others: 15	Embryonal: 16. Alveolar: 21. Pleomorphic: 3. NOS: 2	Localized: 21. Regional: 16. Distant: 5	S+RT+CMT: 54%. S+RT: 4.8%. CMT+RT: 38.1%. RT alone: 2.4%.	5-y OS: 41%. 5-y PFS: 39.7%

## Conclusions

Orbital RMS is a rare but well-documented tumor in pediatrics, where multimodal treatment has significantly improved outcomes. However, in adults, it is a rare, aggressive tumor with a poor prognosis. Our clinical case illustrates the diagnostic and therapeutic challenges in managing these tumors. In the absence of dedicated clinical trials, management remains unstandardized. A collaboration between adult and pediatric oncologists is required. Future research should focus on understanding the molecular differences in adult RMS to develop targeted therapies and improve outcomes.
